# Rapid reviews for health policy and systems decision-making: more important than ever before

**DOI:** 10.1186/s13643-022-01887-7

**Published:** 2022-07-30

**Authors:** Andrea C. Tricco, Sharon E. Straus, Abdul Ghaffar, Etienne V. Langlois

**Affiliations:** 1 Knowledge Translation Program, Li Ka Shing Knowledge Institute, St. Michael’s Hospital, Unity Health Toronto, 209 Victoria Street, East Building, Toronto, M5B 1T8 ON Canada; 2 Epidemiology Division and Institute for Health Management Policy, Management, and Evaluation, Dalla Lana School of Public Health, University of Toronto, Toronto, Ontario Canada; 3 Queen’s Collaboration for Health Care Quality Joanna Briggs Institute Centre of Excellence, Queen’s University, Kingston, Canada; 4 Department of Geriatric Medicine, University of Toronto, Toronto, Ontario Canada; 5 Alliance for Health Policy and Systems Research, Science Division, World Health Organization, Geneva, Switzerland; 6 Partnership for Maternal, Newborn and Child Health, World Health Organization, Geneva, Switzerland

**Keywords:** Rapid reviews, Decision-making, Health systems, Policy, Knowledge synthesis, Evidence synthesis

## Abstract

**Background:**

Due to the explosion in rapid reviews in the literature during COVID-19, their utility in universal health coverage and in other routine situations, there is now a need to document and further advance the application of rapid review methods, particularly in low-resource settings where a scarcity of resources may preclude the production of a full systematic review. This is the introductory article for a series of articles to further the discussion of rapid reviews for health policy and systems decision-making.

**Main body:**

The series of papers builds on a practical guide on the conduct and reporting of rapid reviews that was published in 2019. The first paper provides an evaluation of a rapid review platform that was implemented in four centers in low-resource settings, the second paper presents approaches to tailor the methods for decision-makers through rapid reviews, the third paper focuses on selecting different types of rapid review products, and the fourth pertains to reporting the results from a rapid review.

**Conclusion:**

Rapid reviews have a great potential to inform universal health coverage and global health security interventions, moving forward, including preparedness and response plans to future pandemics. This series of articles will be useful for both researchers leading rapid reviews, as well as decision-makers using the results from rapid reviews.

## Background

Knowledge synthesis is an important tool that can be used by decision-makers, such as patient partners, healthcare providers, and policymakers, to enhance evidence-informed decision-making. Rapid reviews have emerged as a resource-efficient way to conduct knowledge synthesis that can provide evidence in a more timely and relevant manner than other forms of knowledge synthesis [1–6], such as a systematic review. The most recent definition of a rapid review put forth by the Cochrane Rapid Review Methods Group for organizations that only conduct knowledge synthesis for decision-makers is as follows: “A rapid review is a form of knowledge synthesis that accelerates the process of conducting a traditional systematic review through streamlining or omitting various methods to produce evidence for stakeholders in a resource-efficient manner [7].” This definition advances previously proposed definitions for rapid reviews, as it focuses on providing timely evidence to decision-makers. The definition currently does not differentiate between the topic that the rapid review is focused on—such as health services or effectiveness research—and as such, can be applied to all topics.

According to the Cochrane Rapid Review Methods Group, rapid reviews are a demand-driven product [8]. Through a rapid review, the evidence is contextualized for decision-makers, improving the uptake of research results, and leading to an increased impact on decision-making [6, 9]. Furthermore, it is hypothesized that the use of knowledge synthesis products that are relevant to decision-maker needs will reduce research waste [10].

A key approach to further increasing the uptake of rapid reviews includes engaging decision-makers at onset and throughout the entire rapid review process. The intention is for the decision-maker to become a member of the research team using an integrated knowledge generation and uptake approach [11–13]. This strategy is often called “co-production” or “co-creation” of research and is hypothesized to have a much greater impact than simply engaging decision-makers at certain stages of the knowledge synthesis process [14].

Rapid reviews have demonstrated great utility during urgent and emergent situations [15]. Indeed, more than 3000 rapid reviews (and counting!) were conducted during the COVID-19 pandemic [16]. Also, rapid reviews have become an efficient approach to tackling universal health coverage policy and systems decision-making [17]. Now more than ever rapid reviews have become prominent for decision-making.

Previously, there was a dearth of methodological guidance on the conduct of rapid reviews, particularly for health policy and systems research. As such, we published a practical guide on the conduct and reporting of rapid reviews [15]. Due to the explosion in rapid reviews in the literature during COVID-19, as well as their utility in universal health coverage [17, 18] and in other routine situations [19, 20], there is now a need to document and further advance the application of rapid review methods. This is particularly important in low-resource settings, where a scarcity of resources may preclude the production of a full systematic review. Moreover, rapid reviews are critical to answer complex policy and systems questions [21]. To address this need, we have written this series of articles to further the discussion of rapid reviews for health policy and systems decision-making. It is hoped that this series of articles will be useful for both researchers leading rapid reviews, as well as decision-makers using the results from rapid reviews.

## Main text

### First paper

The first paper in the series begins with assessing rapid review centers in low- and middle-income countries (LMICs), supported by the Alliance for Health Policy and Systems Research (HPSR), World Health Organization (WHO), through the Embedded Rapid Review (ERA) initiative [22]. This initiative supported several platforms to conduct rapid reviews for decision-making and included both researcher and decision-maker participants. Sixteen participants in this initiative were interviewed. The interviews identified four themes that supported the use of rapid reviews in policy and systems decision-making: (1) organizational structural arrangements of the platform, (2) management of the rapid review process, (3) rapid reviews as the immediate policy-relevant outputs, and (4) the engagement process. This paper provides guidance for rapid review centers in LMICs, as well as insight into how to increase the impact and relevance of rapid reviews.

### Second paper

The second paper focuses on ways that researchers can tailor rapid review methods to suit the needs of decision-makers [23]. Suggestions to expedite the knowledge synthesis process include using a team experienced in knowledge synthesis, engaging the decision-makers who commissioned the review from project onset and throughout its conduct, and drafting a clear protocol for the rapid review methods. For the literature search, strategies, such as limiting by date or language, as well as using a staged approach to searching (e.g., searching for systematic reviews then randomized trials then observational studies sequentially) are described. Other methods, such as using a single reviewer for steps of the review process (e.g., screening, data abstraction, methodological quality appraisal), use of automation tools in the review process, and providing a short descriptive summary of the evidence, are considered.

### Third paper

The third paper focuses on the process of selecting a rapid review approach from three different types of rapid reviews (annotated bibliographies, thematic summaries, and rapid syntheses) with varying timelines to address complex policy questions [24]. Two stages are discussed; the first is scoping the literature, which involves discussions with the decision-makers who requested the rapid review and conducting literature searches to conceptually map the evidence. The second stage is selecting an optimal approach, which includes additional consultation with decision-makers to refine the question and tailor the methodological approach. Other considerations discussed to guide the selection of a method include the breadth and depth of the literature, time required for the rapid review, and whether a static or evolving conceptual framework can be used to guide the rapid review conceptualization.

### Fourth paper

The final paper in the series provides suggestions on how to report the results from a rapid review [25]. The paper recommends considering the balance between providing sufficient details on the research process with the level of detail requested by the decision-maker, as well as the time and resources available to report the rapid review findings. Suggestions, such as the use of publications or conference presentations to establish clear messaging, crafting the key messages from the review findings with decision-makers, having ongoing engagement with decision-makers throughout the rapid review process, and use of different products for different types of decision-makers, are discussed. The use of pre-prints as a dissemination strategy for rapid reviews is also discussed, which gained more prominence during the COVID-19 pandemic. Finally, gauging impact and reach using dissemination measures and bibliometrics is suggested.

## Conclusions

Several methodological challenges regarding the conduct of rapid reviews have been highlighted in this series of papers, as well as in a recent paper that was published specifically on rapid reviews within the COVID-19 context [26]. We suggest using the Alliance HPSR/WHO guide [17] along with the updated guidance here to tailor rapid review methods for decision-making. In light of the pandemic, rapid reviews have a strategic role to play in supporting evidence-informed policymaking. Rapid reviews have a great potential to inform universal health coverage and global health security interventions, moving forward, including preparedness and response plans to future pandemics.

## Data Availability

The datasets used and/or analyzed during the current study are available from the corresponding author on reasonable request.

## Abbreviations

LMICs: Low- and middle-income countries
HPSR: Alliance for Health Policy and Systems Research
WHO: World Health Organization
ERA: Embedded Rapid Review initiative
